# Senescence-associated gene signatures predict survival in lung cancer: a multi-cohort analysis

**DOI:** 10.1007/s11357-025-01894-1

**Published:** 2025-09-27

**Authors:** Zoltán Ungvári, Otília Menyhart, Alberto Ocana, Mónika Fekete, Andrea Lehoczki, Balázs Győrffy

**Affiliations:** 1 Vascular Cognitive Impairment, Neurodegeneration and Healthy Brain Aging Program, Department of Neurosurgery, University of Oklahoma Health Sciences Center, Oklahoma City, OK USA; 2 Stephenson Cancer Center, University of Oklahoma, Oklahoma City, OK USA; 3 Oklahoma Center for Geroscience and Healthy Brain Aging, University of Oklahoma Health Sciences Center, Oklahoma City, OK USA; 4 Department of Health Promotion Sciences, College of Public Health, University of Oklahoma Health Sciences Center, Oklahoma City, OK USA; 5 International Training Program in Geroscience, Doctoral College/Institute of Preventive Medicine and Public Health, Semmelweis University, Budapest, Hungary; 6 Cancer Biomarker Research Group, Institute of Molecular Life Sciences, Hungarian Research Network, Magyar Tudósok Körútja 2, 1117 Budapest, Hungary; 7 National Laboratory for Drug Research and Development, Magyar Tudósok Körútja. 2. 1117, Budapest, Hungary; 8 Department of Bioinformatics, Semmelweis University, 1094 Budapest, Hungary; 9 Experimental Therapeutics in Cancer Unit, Instituto de Investigación Sanitaria San Carlos (IdISSC), and, CIBERONC , Madrid, Spain; 10 INTHEOS-CEU-START Laboratory, Facultad de Medicina, Universidad CEU San Pablo, 28668 Boadilla del Monte, Madrid, Spain; 11 Institute of Preventive Medicine and Public Health, Semmelweis University, Budapest, Hungary; 12 Fodor Center for Prevention and Healthy Aging, Semmelweis University, Budapest, Hungary; 13 Doctoral College, Health Sciences Division, Semmelweis University, Budapest, Hungary; 14 Department of Biophysics, Medical School, University of Pecs, Pecs, 7624 Hungary

**Keywords:** Senescence, Lung cancer, Prognosis, Aging, Biomarkers, Gene expression, Gero-oncology, SenMayo, SASP, Senolytics

## Abstract

Lung cancer is a leading cause of cancer-related mortality worldwide and is predominantly diagnosed in older adults, underscoring the need to explore aging-related biological mechanisms that influence disease progression and prognosis. Cellular senescence, a hallmark of aging, plays a dual role in cancer by contributing to both tumor suppression and tumor promotion through its influence on tumor growth, modulation of the tumor microenvironment, the senescence-associated secretory phenotype (SASP), and response to therapy. In this study, we evaluated the prognostic significance of senescence-related gene expression in lung cancer using three independent gene signatures, including the SenMayo gene set and two additional curated lists. Transcriptomic and clinical data from publicly available datasets were analyzed using Cox regression, Kaplan–Meier survival analysis, and multivariate modeling. All three senescence signatures were significantly associated with overall survival, with the SenMayo signature showing the most robust and consistent prognostic power. Notably, higher expression of senescence-associated genes was associated with improved survival in the overall lung cancer cohort and in lung adenocarcinoma, while a more heterogeneous pattern emerged in squamous cell carcinoma. Although hazard ratios varied among the gene sets, their broadly concordant associations with clinical outcomes highlight the biological relevance and context dependence of senescence in lung cancer. These findings suggest that senescence-associated gene expression may serve as a valuable prognostic biomarker and offer mechanistic insights into tumor behavior. Our results contribute to the growing body of gero-oncology research and emphasize the need for tumor-specific exploration of aging-related processes in cancer.

**Supplementary Information:**

The online version contains supplementary material available at 10.1007/s11357-025-01894-1.

## Introduction

Lung cancer remains one of the leading causes of cancer-related mortality worldwide, accounting for nearly 1.8 million deaths annually [1–3]. Despite advances in early detection and treatment, the overall 5-year survival rate for lung cancer remains dismal, especially in patients diagnosed at advanced stages [4]. Non-small cell lung cancer (NSCLC), which comprises the majority of Lung cancer cases, is often diagnosed in older adults, with the median age at diagnosis exceeding 70 years [5]. This strong age-related incidence pattern highlights the need to explore the underlying biological mechanisms that link aging and lung tumorigenesis.

The emerging field of geroscience offers a novel framework for understanding lung cancer pathogenesis by examining how fundamental mechanisms of aging contribute to disease development and progression [6]. One such hallmark of aging is cellular senescence [7–10], a stress-induced, stable cell cycle arrest that is accompanied by a pro-inflammatory and tissue-remodeling phenotype termed the senescence-associated secretory phenotype (SASP). While senescence acts as a tumor-suppressive barrier by limiting the propagation of damaged or transformed cells, persistent senescent cells in the tissue microenvironment may paradoxically promote tumor progression via chronic inflammation, extracellular matrix remodeling, and immune modulation [7–10].

Recent studies have increasingly recognized the dual role of senescence in cancer biology, and gene expression signatures associated with senescence have been proposed as potential biomarkers of tumor behavior and therapeutic vulnerability [7–17]. The *SenMayo* gene set, a curated signature derived from senescent cells across tissues and species [18], has been validated as a tool to detect senescence-associated transcriptional activity and assess its impact on clinical outcomes [19]. Previous studies using this gene set in colorectal, breast, and myeloma cohorts have demonstrated its robust association with survival, independent of standard clinical parameters [19].

Given the strong biological rationale and the unmet need for prognostic biomarkers in lung cancer, we aimed to evaluate the prognostic significance of a senescence-associated gene signature in a large cohort of lung cancer patients. Leveraging publicly available transcriptomic datasets and standardized bioinformatics pipelines, we assessed the association between *SenMayo*-derived gene expression patterns and overall survival. Our hypothesis was that senescence-related gene expression would stratify patients according to survival outcomes and provide mechanistic insight into age-related alterations in the tumor microenvironment. This work contributes to the growing field of gero-oncology and supports the integration of aging biology into cancer prognostication and therapeutic decision-making.

## Methods

### Dataset identification and inclusion criteria

Transcriptome-wide gene expression datasets relevant to lung cancer were retrieved from the Gene Expression Omnibus (GEO) (https://www.ncbi.nlm.nih.gov/geo/) and The Cancer Genome Atlas (TCGA) databases. To ensure Sufficient statistical power and clinical relevance, datasets were included only if they contained at least 30 primary lung tumor samples, included corresponding clinical outcome data, and were generated using Affymetrix microarray platforms GPL96, GPL570, or GPL571. These platforms were selected due to their consistent probe architecture, which profiles 22,277 transcripts using identical oligonucleotide sequences. This compatibility enables robust cross-study integration and minimizes platform-related variability.

### Data normalization and quality control

All available raw CEL files underwent standardized preprocessing to ensure high-quality expression measurements across datasets. Data normalization was performed using the MAS5 algorithm, selected for its well-established concordance with RT-PCR validation and its ability to normalize samples independently of dataset composition [20]. To further minimize technical variance and control for batch effects, we applied a secondary scaling normalization step, adjusting the mean signal intensity of the 22,277 shared probes to a uniform target value of 1000 across all arrays. To maintain methodological consistency, only those probes shared with the GPL96 array platform were retained, thereby excluding platform-specific extensions present in GPL570 [21]. To ensure probe-level accuracy, we used the JetSet algorithm to identify the most reliable probe set representing each gene based on criteria for specificity, consistency, and dynamic range. Redundant samples (e.g., technical replicates with identical expression profiles) were filtered, retaining only the first occurrence. Comprehensive quality control metrics were then applied, including assessments of background signal, noise levels, and the detection rate (the percentage of present calls that were detected). Additional quality validation included the performance of spike-in controls (bioBCD) and the 3′/5′ expression ratios of the housekeeping genes GAPDH and ACTB to assess RNA quality and integrity. Only samples that passed all quality control checks or fell within the 95% confidence interval of key continuous metrics were retained for downstream analyses. Outliers—defined as samples failing any critical QC parameter—were excluded to ensure the reliability and interpretability of subsequent survival and expression analyses. This harmonized preprocessing framework [22] resulted in a high-quality, integrative lung cancer gene expression dataset suitable for robust prognostic modeling and statistical evaluation.

### Senescence signatures

To evaluate the prognostic relevance of cellular senescence in lung cancer, we compared senescence-associated gene sets derived from prior studies. The SenMayo gene set was initially curated and validated by Saul et al. as a canonical senescence signature [18]. This set encompasses genes consistently upregulated in senescent cells across multiple species and tissues, capturing key elements of the senescence program, including cell cycle arrest, DNA damage response, mitochondrial dysfunction, and the senescence-associated secretory phenotype (SASP). Of the 125 original SenMayo genes, 122 were identified in our transcriptomic dataset after probe remapping using the JetSet algorithm on the GPL96 platform (Supplemental Table 1). A senescence score was computed as a weighted average of gene expression, where weights were derived from univariate hazard ratios (HRs): genes with HR > 1 were assigned negative weights (indicating risk association), and those with HR < 1 were assigned positive weights (suggesting a protective role).

**Table 1:** Summary of gene array datasets comprising the integrated Lung cancer gene expression database. The database contains tumor samples derived from 17 publicly available transcriptomic datasets, including 16 datasets from the Gene Expression Omnibus (GEO) repository and one dataset from The Cancer Genome Atlas (TCGA)

Dataset	Publication PMID	GPL	Sample size (n)
GSE102287	29196495	GPL570	66
GSE14814	20823422	GPL96	90
GSE157011	32717408	GPL570	235
GSE19188	20421987	GPL570	156
GSE29013	21742808	GPL570	55
GSE30219	23698379	GPL570	307
GSE31210	23028479	GPL570	246
GSE3141	16273092	GPL570	111
GSE31908	NA	GPL570, GPL96	40
GSE37745	23032747 29112949 26608184 33576873 35574381	GPL570	196
GSE43580	23966112	GPL570	150
GSE4573	16885343	GPL96	130
GSE50081	24305008	GPL570	181
GSE68465	18641660	GPL96	462
GSE77803	NA	GPL570	156
GSE8894	19010856	GPL570	138
TCGA	25079552	NA	133

The Li 2025 gene signature [23] was derived from a larger set of pan-cancer endothelial senescence-associated genes originally defined by Wu et al. (2023) [24]. The original EC.SENESCENCE.SIG gene set (*n* = 102) was constructed by integrating 18 single-cell RNA-seq datasets across 15 tumor types, with a specific focus on endothelial cells [24]. Li et al. refined this set by performing univariate Cox regression analysis in the TCGA-LUAD cohort, selecting genes significantly associated with overall survival [23]. The selection yielded a 32-gene prognostic model, of which 30 genes were present in our dataset (Supplemental Table 1) [23]. In the original study, this model effectively stratified patients with LUAD into high- and low-risk groups characterized by distinct molecular and immune features [23]. To align with our SenMayo-based analysis, we recalculated the Li 2025 senescence score as a weighted average of gene expression, applying hazard ratio-based weights using the same methodology.

Third, we also evaluated the prognostic performance of the full EC.SENESCENCE.SIG gene set developed by Wu et al., consisting of 102 endothelial senescence-associated genes [24], of which 97 were available in our dataset (Supplemental Table 1). Consistent with our analytical framework, a Weighted average expression score was calculated for this signature based on gene-specific hazard ratios. However, given that the Li 2025 senescence signature was optimized explicitly for lung adenocarcinoma and represents a prognostically refined Subset of the Wu 2023 senescence gene set, we prioritized the SenMayo and Li 2025 signatures for inclusion in the main manuscript. The analysis of the Wu 2023 senescence signature is provided in the Supplementary Materials for completeness.

All three signatures were tested for prognostic relevance in lung cancer datasets using Kaplan–Meier analysis and Cox proportional hazards modeling. Signature performance was assessed based on survival stratification, statistical significance, and hazard ratios.

### Univariate survival analysis

To evaluate the prognostic relevance of senescence-associated gene expression in lung cancer, we employed the Kaplan–Meier plotter platform [25, 26]. Univariate Cox proportional hazards regression was performed to assess the association between each senescence gene signature and overall survival (OS) and first progression (FP) in patients with lung cancer. To reduce bias introduced by arbitrary cutoff selection, we analyzed expression values across the interquartile range (25th–75th percentiles). Optimal cutoff points were identified automatically based on the most statistically significant split, and Kaplan–Meier survival curves were generated to illustrate survival differences between high- and low-expression groups visually. To adjust for multiple hypothesis testing, *p*-values were corrected using the Benjamini–Hochberg method, and genes were considered statistically significant at a false discovery rate (FDR) below 10% [27].

### Multivariate survival analysis

Multivariate Cox regression analysis was conducted to determine whether the prognostic impact of the senescence gene signatures was independent of key clinical variables in lung cancer. Clinical covariates included gender, tumor stage, smoking history, and histological subtype. To address variability in data completeness across clinical annotations, each covariate was analyzed in a separate two-variable model alongside the senescence signature (e.g., signature + histology; signature + gender). This approach ensured the maximal inclusion of samples for each comparison while still assessing the additive prognostic value of the signature. Hazard ratios (HRs), 95% confidence intervals (CIs), and p-values were reported for each model to quantify the relative impact of senescence-associated gene expression on survival outcomes in lung cancer.

## Results

### The lung cancer gene array database

**Fig. 1:**
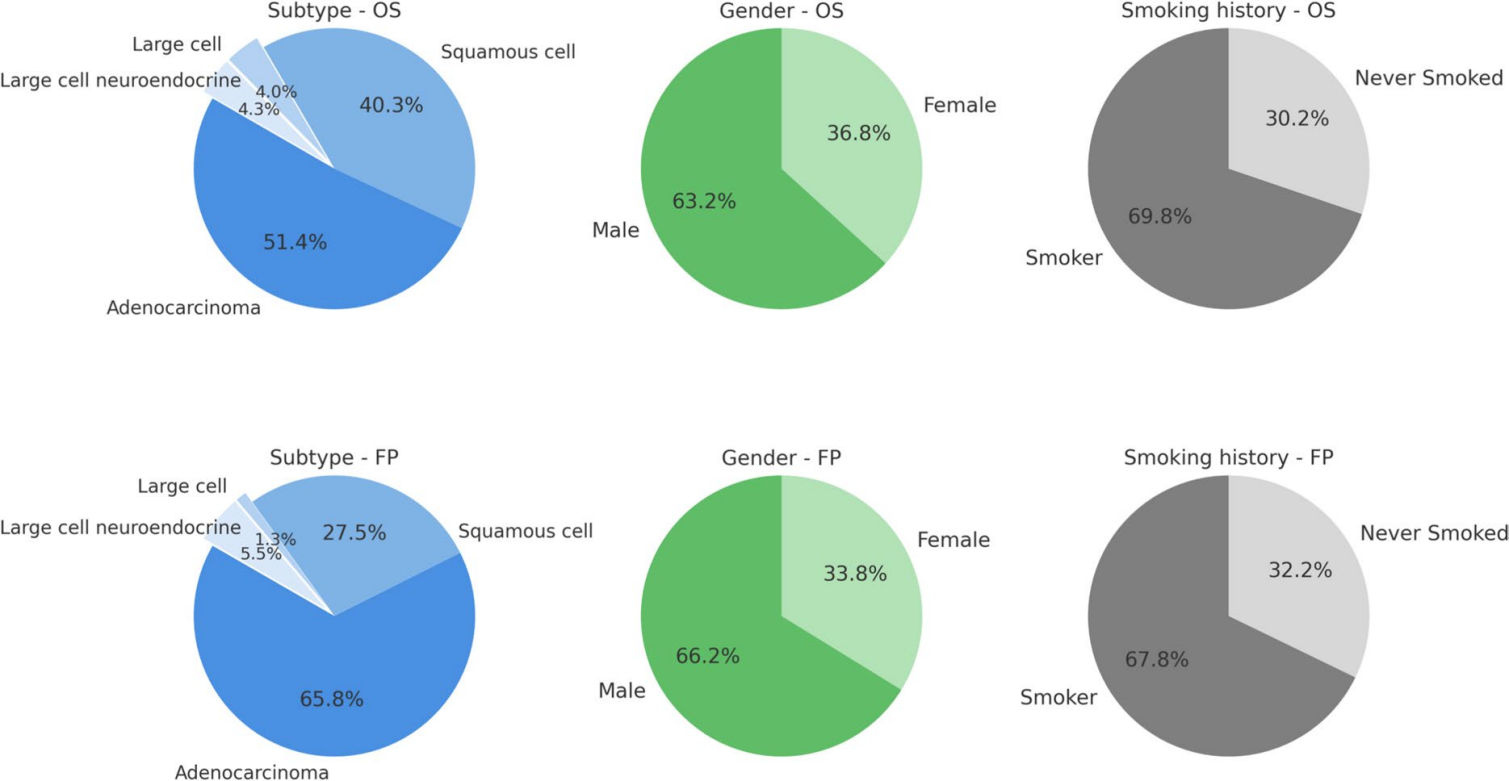
Distribution of clinical characteristics in the overall survival (OS; *n* = 1,406) and first progression (FP; *n* = 870) lung cancer cohorts. Pie charts display the proportion of patients with adenocarcinoma, squamous cell carcinoma, large cell carcinoma, and large cell neuroendocrine carcinoma. Percentages reflect only patients with complete histological subtype annotations and may differ slightly from those reported in the main text, which are calculated based on the full cohort

The comprehensive, integrated Lung cancer database comprises 2,852 tumor samples derived from 17 primary datasets (Table 1). To evaluate lung cancer prognosis, we utilized two distinct patient cohorts: one with overall survival outcomes (OS; *n* = 1,406) and another with information on first progression (FP; *n* = 870) (Table 2). In the OS lung cancer dataset, adenocarcinoma (LUAD) accounted for nearly half of all cases (670 patients, 47.7%), followed by squamous cell carcinoma (LUSC) with 526 patients (37.4%). Less common histological subtypes included large cell carcinoma (52 patients, 3.7%) and large cell neuroendocrine carcinoma (56 patients, 4.0%) (Fig. 1).

**Table 2:** Clinical and demographic characteristics of lung cancer patient populations included in the overall survival (OS) and first progression (FP) cohorts

Lung cancer, OS cohort		Number of patients	Lung cancer, FP cohort		Number of patients
total		1406	total		870
*subtype*	adenocarcinoma	670	*subtype*	adenocarcinoma	526
	squamous cell cc	526		squamous cell cc	220
	large cell cc	52		large cell cc	10
	large cell neuorend. cc	56		large cell neuorend. cc	44
*AJCC STAGE T:*	1	220	*AJCC STAGE T:*	1	212
	2	190		2	185
	3	33		3	29
	4	21		4	20
*AJCC STAGE N:*	0	324	*AJCC STAGE N:*	0	313
	1	104		1	100
	2	30		2	27
*AJCC STAGE M:*	0	459	*AJCC STAGE M:*	0	442
	1	8		1	6
*sex*	female	476	*sex*	female	294
	male	819		male	576
*smoking*	never smoked	143	*smoking*	never smoked	141
	smoker	330		smoker	297

Abbreviations: OS, overall survival; FP, first progression; cc., carcinoma; large cell neuorend. cc., large cell neuroendocrine carcinoma

The FP dataset mirrored this histological distribution, although proportions varied slightly. Regarding tumor size classification (AJCC T-stage), the majority of cases in both the OS and FP cohorts were classified as T1 or T2 tumors, with fewer patients presenting advanced-stage (T3 or T4) tumors. Similarly, lymph node involvement (AJCC N-stage) was predominantly absent in both datasets (N0), though a considerable proportion had evidence of regional lymphatic spread (N1/N2). For distant metastasis (AJCC M-stage), most patients in both cohorts had localized disease without distant metastases (M0), aligning with the predominantly localized nature of the disease presentation. Demographically, there was a male predominance in both cohorts, consistent with established epidemiological patterns of lung cancer. Additionally, the majority of patients reported a history of smoking, although a notable proportion were never-smokers (OS: 143 patients, 10.2%; FP: 141 patients, 16.2%), highlighting the relevance of non-smoking-associated etiologies in lung cancer (Fig. 1).

### Prognostic performance of the SenMayo signature

**Fig. 2:**
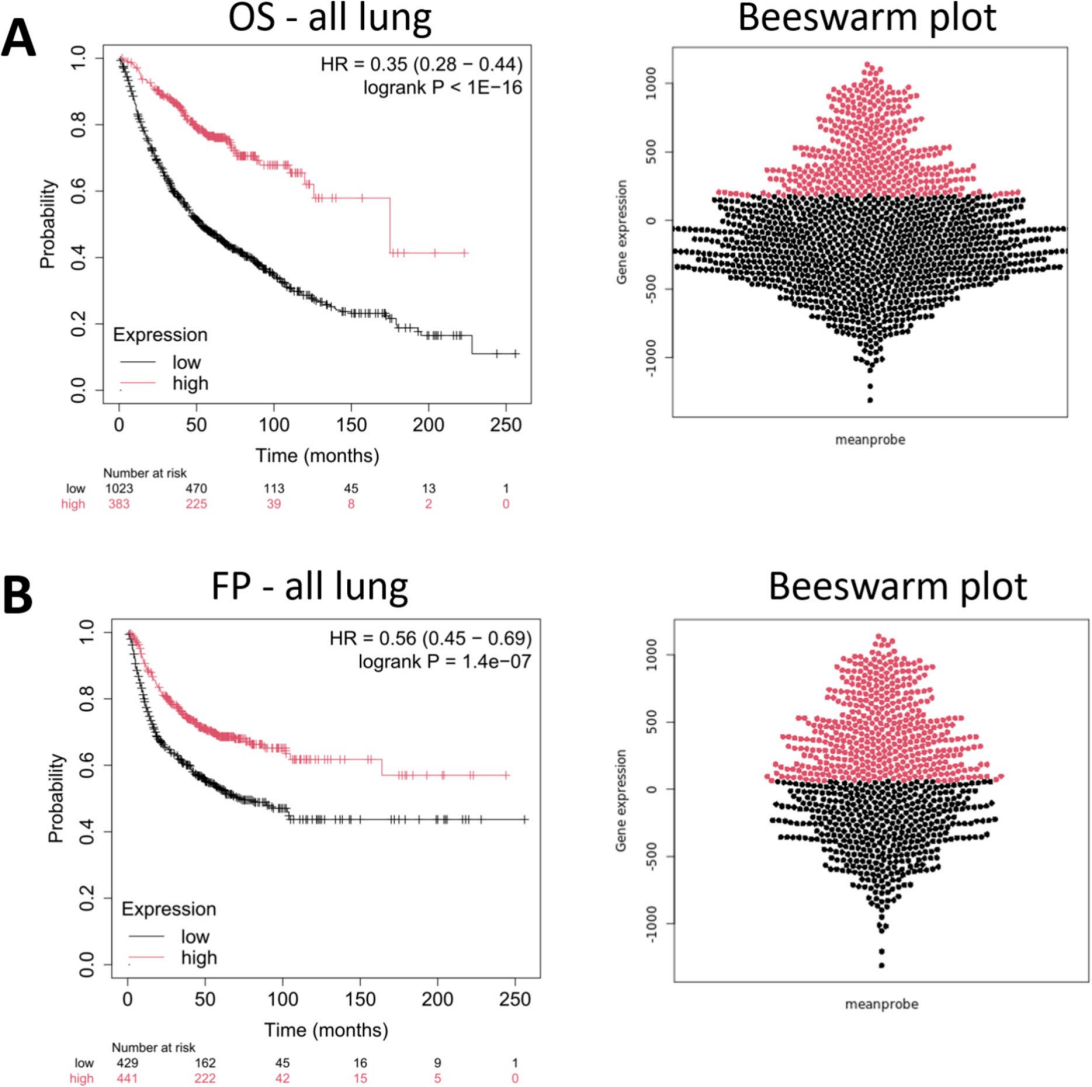
Kaplan–Meier plots illustrating the association between SenMayo gene signature expression and clinical outcomes in lung cancer patients. Patients were dichotomized into high- and low-expression groups based on the weighted mean SenMayo expression scores. **A**) In the overall survival (OS) cohort (*n* = 1,406), higher SenMayo expression was significantly associated with improved survival. The beeswarm plot (right panels) illustrates the distribution of senescence scores across individual patients, with red indicating high-expression and black indicating low-expression groups. **B**) In the first progression (FP) cohort (*n* = 870), the high expression also predicted delayed progression. *Abbreviations*: OS, overall survival; FP, first progression; all lung, comprehensive lung cancer cohort

**Fig. 3:**
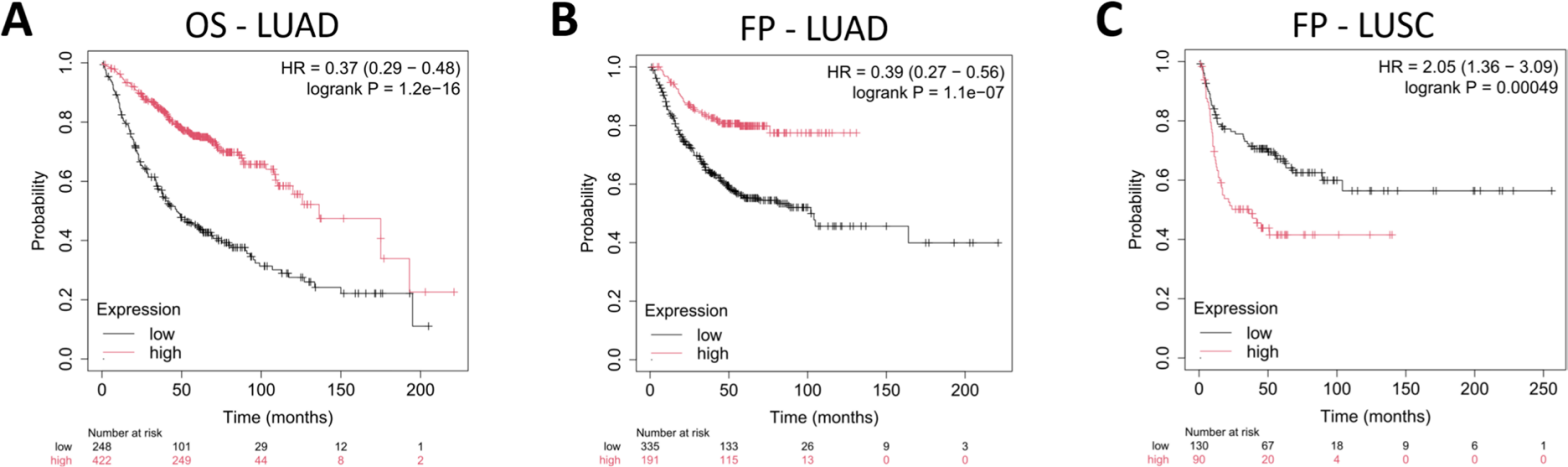
Prognostic significance of the SenMayo senescence gene signature in lung cancer subtypes. **A**) In lung adenocarcinoma (LUAD), high SenMayo expression was associated with improved overall survival (OS). **B**) In LUAD, high expression also predicted delayed first progression (FP). **C**) In contrast, in the lung squamous cell carcinoma (LUSC) subgroup, high SenMayo expression was significantly associated with worse FP. *Abbreviations:* OS, overall survival; FP, first progression; LUAD, lung adenocarcinoma; LUSC, lung squamous cell carcinoma

We evaluated the association between the weighted mean expression of the SenMayo gene signature [18] and clinical outcomes in the Lung Cancer Gene Array Database. The analysis demonstrated a robust prognostic value of the signature in both the overall survival (OS) and first progression (FP) datasets. In the combined lung cancer cohort (all lung), high SenMayo senescence signature expression was associated with significantly improved OS (HR = 0.35, 95% CI = 0.28–0.44, log-rank *p* = 1e-16) and FP (HR = 0.56, 95% CI = 0.45–0.69, log-rank *p* = 1.4e-07) (Fig. 2A, B). Stratification by histological subtype revealed a pronounced prognostic effect in LUAD, where a high SenMayo signature expression was linked to prolonged OS (HR = 0.37, 95% CI = 0.29–0.48, log-rank *p* = 1.2e-16) and FP (HR = 0.39, 95% CI = 0.27–0.56, log-rank *p* = 1.1e-07) (Fig. 3A, B).

Conversely, in LUSC, the SenMayo signature exhibited prognostic significance specifically for FP but not OS, with high expression associated with earlier first progression (HR = 2.05, 95% CI = 1.36–3.09, log-rank *p* = 0.00049), suggesting a histology-specific role of senescence-associated gene expression in lung cancer progression (Fig. 3C). Our findings suggest that the SenMayo senescence signature may identify distinct biological pathways that govern disease progression and survival outcomes in various histological contexts.

### Prognostic performance of the Li 2025 senescence signature

Second we assessed the prognostic significance of the Li et al. 2025 senescence-associated gene signature [23]—derived initially from endothelial senescence analysis in LUAD—across multiple lung cancer subtypes in our Lung Cancer Gene Array Database. A Weighted average expression score was computed based on the 30 available of the original 32 genes (Supplemental Table 1).

**Fig. 4:**
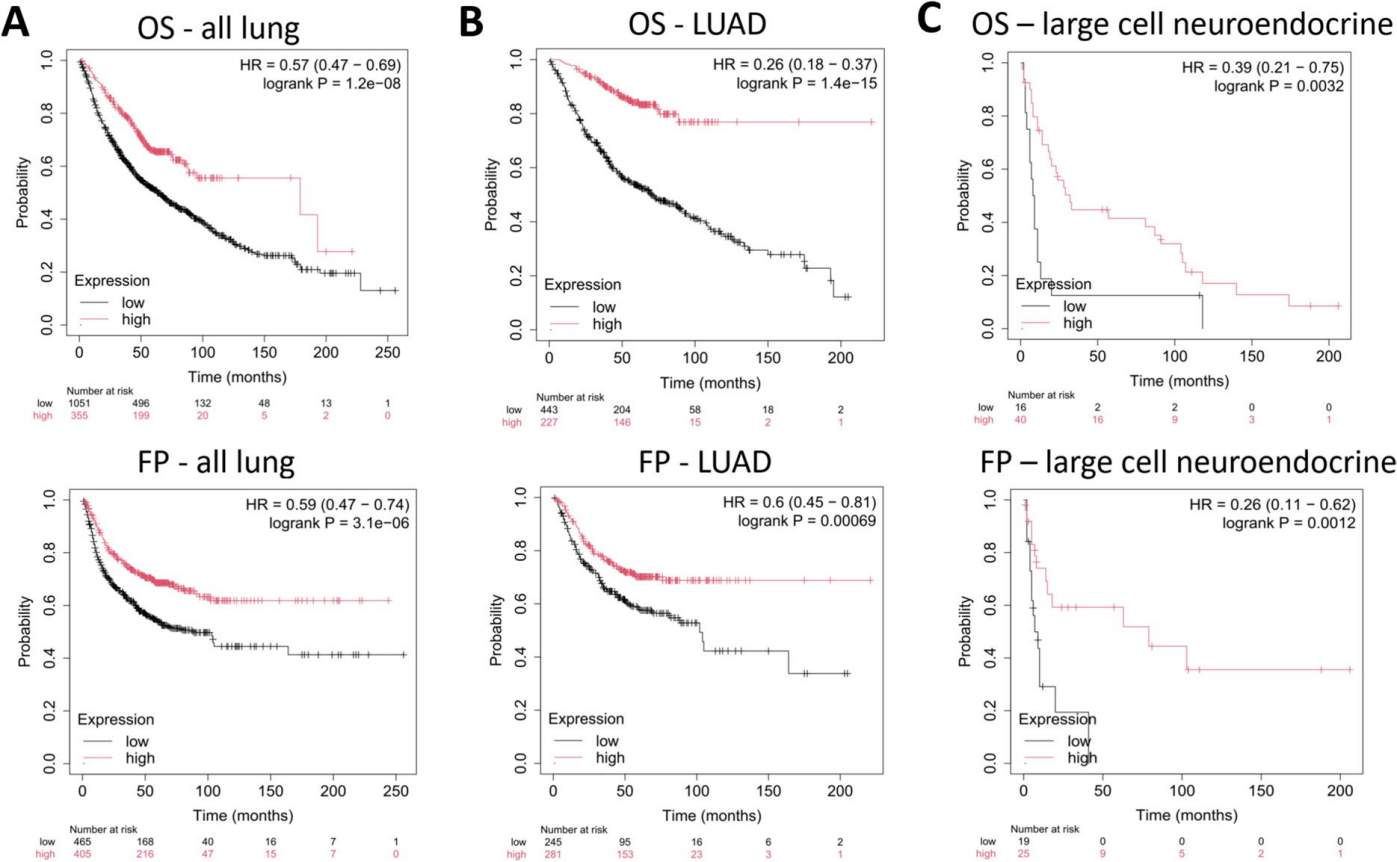
Prognostic significance of the Li 2025 senescence gene signature in lung cancer. **A**) In the overall lung cancer cohort (all lung), high signature expression was associated with improved overall survival (OS) and delayed first progression (FP). **B**) Stratified analyses demonstrated strong prognostic power in lung adenocarcinoma (LUAD), where elevated gene signature expression correlated with prolonged OS and FP **C**). The signature also showed significant prognostic relevance in large-cell neuroendocrine carcinoma, where high expression was associated with improved OS and FP. *Abbreviations:* OS, overall survival; FP, first progression; all lung, comprehensive lung cancer cohort; LUAD, lung adenocarcinoma; LUSC, lung squamous cell carcinoma

In the pan-lung cancer cohort (all lung cancer samples), high expression of the signature was associated with significantly improved OS (HR = 0.57, 95% CI = 0.47–0.69, log-rank *p* = 1.2e-08) and FP (HR = 0.59, 95% CI = 0.47–0.74, log-rank *p* = 3.1e-06) (Fig. 4A). When stratified by histological subtype, the strongest association was observed in LUAD, where high signature expression was linked to markedly better survival: for OS, the hazard ratio was 0.26 (95% CI = 0.18–0.37, log-rank *p* = 1.4e-15), and the FP hazard ratio was 0.6 (95% CI = 0.45–0.81, log-rank *p* = 0.00069), demonstrating the high discriminatory power of the signature in this subtype (Fig. 4B). Moreover, in large-cell neuroendocrine carcinoma, higher expression of the Li 2025 senescence signature was significantly associated with improved OS (HR = 0.39, 95% CI = 0.21–0.75, log-rank *p* = 0.0032) and FP (HR = 0.26, 95% CI = 0.11–0.62, log-rank *p* = 0.0012) at FDR = 10%, suggesting potential clinical utility in this aggressive histological subtype. However, these findings should be interpreted with caution due to the limited sample size (Fig. 4C).

The Wu 2023 signature [24] provided similar results in the pan-cancer and LUAD cohorts (Supplemental Fig. 1). Nevertheless, it did not retain prognostic value in other histologies and did not provide novel information; therefore, it was included in the Supplementary materials.

### Multivariate analysis

To evaluate whether our senescence-associated gene signatures provided prognostic information independent of common clinical variables, we performed multivariate Cox regression analyses. Both the SenMayo and Li (2025) senescence gene signatures independently predicted improved overall survival after adjusting for histology, disease stage, gender, and smoking history (Table 3). These findings support the robustness and clinical relevance of the senescence-related gene signature as an independent prognostic biomarker in patients with lung cancer.

**Table 3:** Multivariate Cox regression analysis evaluating the independent prognostic significance of two senescence-associated gene signatures in lung cancer. Each signature was tested in separate models adjusted for key clinical parameters

**Senescence Signature**	**Clinical Factor Adjusted For**	**HR (95% CI)**	***P*-value**
SenMayo	Histology	0.68 (0.57–0.81)	< 0.001
	Disease Stage	0.55 (0.45–0.68)	< 0.001
	Gender	0.59 (0.50–0.69)	< 0.001
	Smoking History	0.38 (0.25–0.56)	< 0.001
Li (2025)	Histology	0.78 (0.66–0.92)	0.0028
	Disease Stage	0.78 (0.64–0.95)	0.0155
	Gender	0.75 (0.64–0.87)	0.0002
	Smoking History	0.46 (0.31–0.67)	0.0001

Abbreviations: HR, hazard ratio; CI, confidence interval

## Discussion

Our study demonstrates that the expression of senescence-associated genes, as captured by the SenMayo signature, is significantly associated with overall survival in lung cancer. This finding reinforces the emerging view that cellular senescence—a hallmark of aging [28, 29]—plays a critical role not only in the initiation but also in the progression of age-related malignancies [7–10]. The observed association between a senescence gene expression profile and patient survival suggests that senescence-related processes are active within the lung tumor microenvironment and may influence clinical outcomes.

These results are broadly consistent with our previous findings in colorectal cancer, breast cancer, and multiple myeloma [19], where senescence gene expression stratified patients by survival or relapse. However, they also underscore important tumor-specific differences in how senescence contributes to disease biology. For instance, in colorectal cancer, increased senescence-related gene expression correlates with poorer prognosis—likely reflecting the pro-tumorigenic effects of SASP and stromal senescence—whereas in breast cancer and myeloma [19], higher senescence scores are generally protective. In lung cancer, our data suggest a more nuanced picture: while senescence may initially suppress malignant transformation, chronic accumulation of senescent cells might promote immune evasion, angiogenesis, or therapy resistance, depending on the cellular and microenvironmental context [30–38]. This highlights the context-dependent duality of senescence and underscores the need for tumor-specific models of senescence biology [10, 39].

To explore the robustness of senescence-related transcriptional activity as a prognostic marker, we employed three independent senescence gene sets: the widely used SenMayo signature and two additional curated lists based on recent transcriptomic and functional studies. All three signatures showed significant associations with overall survival in at least one of the analyzed cohorts, supporting the overarching concept that senescence captures clinically relevant features of tumor biology. However, the variation in hazard ratios and effect sizes among the signatures suggests that each may emphasize distinct aspects of the senescence program—such as SASP-related inflammation, mitochondrial dysfunction, or the DNA damage response—as well as reflect differences in the cell-type specificity of senescence across the tumor microenvironment, including epithelial [40–42], stromal [43], endothelial [44, 45], or immune compartments [35, 37].

Notably, the SenMayo signature was developed across diverse tissues and species [18], while the other two gene sets may contain genes more specifically enriched in lung-resident or endothelial cells. This biological diversity likely contributes to the observed differences in prognostic performance. Furthermore, limited gene overlap between the lists reinforces the idea that senescence is not a uniform transcriptional program but rather a heterogeneous and context-sensitive process [7]. Despite these differences, the broadly consistent associations with survival support the potential utility of senescence profiling in lung cancer [37]. Future work should aim to refine and integrate these signatures into composite classifiers optimized for lung-specific biology and therapeutic applications.

Importantly, our stratified analyses revealed histology-specific differences in the directionality of the prognostic associations. In lung adenocarcinoma, higher expression of senescence-associated gene signatures was consistently linked to improved overall survival and delayed first progression, suggesting that senescence-related processes may exert a predominantly tumor-suppressive effect in this subtype. In contrast, in lung squamous cell carcinoma, the same signatures—particularly SenMayo—were associated with poorer outcomes, indicating a potential shift toward tumor-promoting effects, possibly driven by pro-inflammatory SASP activity, stromal remodeling, or altered immune interactions. These divergent prognostic trends underscore the context-dependent nature of senescence biology and caution against a uniform interpretation of senescence markers across histologies. Lung adenocarcinoma and lung squamous cell carcinoma differ markedly in smoking prevalence, driver mutation spectra, and immune microenvironment composition, all of which can influence the senescence program. Chronic tobacco exposure, for example, may promote a more pro-inflammatory SASP phenotype in squamous carcinogenesis, whereas in lung adenocarcinoma, senescence programs may act to restrain tumor aggressiveness. Future mechanistic studies should explore the molecular and cellular drivers of this divergence, including differences in tissue architecture, mutational burden, and immune cell infiltration between lung adenocarcinomas and squamous cell carcinomas.

The lung tumor microenvironment presents additional complexity, shaped by chronic inflammation [34, 46, 47], smoking-induced DNA damage [47], and immunosenescence [35, 37, 48, 49]. These factors may affect the abundance and function of senescent cells. For example, smoking may trigger a pro-inflammatory SASP phenotype that fosters tumor progression. Likewise, senescent immune cells [35, 37] or SASP-derived cytokines may contribute to immune exhaustion and therapy resistance. Understanding these interactions will be key to decoding the prognostic and therapeutic implications of senescence in lung cancer.

The therapeutic relevance of our findings is underscored by the emerging field of senescence-targeted interventions. Senolytic agents—such as navitoclax (a Bcl-2/Bcl-xL inhibitor) [50–54] and dasatinib/quercetin [55–60]—have shown promise in preclinical lung cancer models as well as in clinical studies, likley by clearing therapy-induced senescent cells and reducing residual disease. In parallel, senostatics that suppress SASP signaling without eliminating senescent cells may help mitigate the pro-tumorigenic effects of the microenvironment [61]. Since many standard therapies induce senescence in both tumor and stromal cells, combining them with senolytics may offer synergistic benefits [34, 41, 42]. Further studies are needed to identify which lung cancer subtypes are most susceptible to these strategies and whether senescence signatures can guide treatment selection.

Several limitations warrant consideration. The retrospective design and reliance on publicly available transcriptomic datasets limit control over treatment heterogeneity and confounders. Additionally, while the SenMayo signature reflects aggregate senescence-related gene expression, it does not resolve cell-type specificity within the tumor microenvironment. Future research using spatial transcriptomics or single-cell RNA sequencing could clarify how senescence manifests across tumor, stromal, endothelial, and immune compartments. Unfortunately, detailed mutational data (e.g., KRAS, EGFR, BRAF, ALK, ROS1, NTRK) were not consistently available across the public datasets we analyzed, precluding robust stratified analyses by oncogenic driver status. Integration of mutation profiles with senescence-associated gene expression in future multi-omic studies could clarify whether senescence interacts with specific oncogenic pathways to influence prognosis. Finally, although our findings demonstrate a prognostic association, functional validation is needed to establish causality and mechanistic underpinnings.

From a translational perspective, incorporation of SASP metrics into clinical decision-making will require assays that are robust, rapid, and cost-effective. One feasible approach would be the development of small, qPCR-based panels targeting the most prognostically relevant senescence-associated genes identified in this and prior studies, enabling integration into routine pathology workflows. Such panels could complement existing histopathological and molecular markers to refine prognostic stratification.

In conclusion, this study supports the prognostic significance of senescence-associated gene expression in lung cancer and highlights the heterogeneous nature of senescence across tumor types. Integrating senescence biomarkers into clinical decision-making could improve risk stratification and help identify patients who may benefit from emerging senescence-targeted therapies. These findings add to the growing body of gero-oncology research and point toward new opportunities for tailoring cancer care in the context of aging.

## Supplementary Information

Below is the link to the electronic supplementary material.

Supplementary file 1 Supplemental Fig. 1. Prognostic value of the Wu 2023 senescence gene signature in lung cancer. A) In the combined lung cancer cohort ("all lung"), high expression of the senescence signature was significantly associated with prolonged overall survival (OS) and delayed first progression (FP). B) Subtype-specific analyses revealed prognostic performance in lung adenocarcinoma (LUAD), where elevated signature expression correlated with improved OS and FP. *Abbreviations:* OS, overall survival; FP, first progression; all lung, comprehensive lung cancer cohort; LUAD, lung adenocarcinoma; LUSC, lung squamous cell carcinoma. Supplemental Table 1. Gene lists of the senescence signatures. (DOCX 438 KB)

## Data Availability

NA.
