# Supplementary material for: Senescence-associated gene signatures predict survival in lung cancer: a multi-cohort analysis
Source: GeroScience. 2025 Sep 27;48(1):577–90. doi: 10.1007/s11357-025-01894-1 (PMC12972208; doi:10.1007/s11357-025-01894-1)
Supplement: Supplementary file 1 — Supplemental Fig. 1. Prognostic value of the Wu 2023 senescence gene signature in lung cancer. A) In the combined lung cancer cohort ("all lung"), high expression of the senescence signature was significantly associated with prolonged overall survival (OS) and delayed first progression (FP). B) Subtype-specific analyses revealed prognostic performance in lung adenocarcinoma (LUAD), where elevated signature expression correlated with improved OS and FP. Abbreviations: OS, overall survival; FP, first progression; all lung, comprehensive lung cancer cohort; LUAD, lung adenocarcinoma; LUSC, lung squamous cell carcinoma. Supplemental Table 1. Gene lists of the senescence signatures. (DOCX 438 KB) [file 11357_2025_1894_MOESM1_ESM.docx]

**SUPPLEMENTARY MATERIALS**

**Supplemental Figure 1. Prognostic value of the Wu 2023 senescence gene signature in lung cancer.** A) In the combined lung cancer cohort ("all lung"), high expression of the senescence signature was significantly associated with prolonged overall survival (OS) and delayed first progression (FP). B) Subtype-specific analyses revealed prognostic performance in lung adenocarcinoma (LUAD), where elevated signature expression correlated with improved OS and FP. *Abbreviations:* OS, overall survival; FP, first progression; all lung, comprehensive lung cancer cohort; LUAD, lung adenocarcinoma; LUSC, lung squamous cell carcinoma.


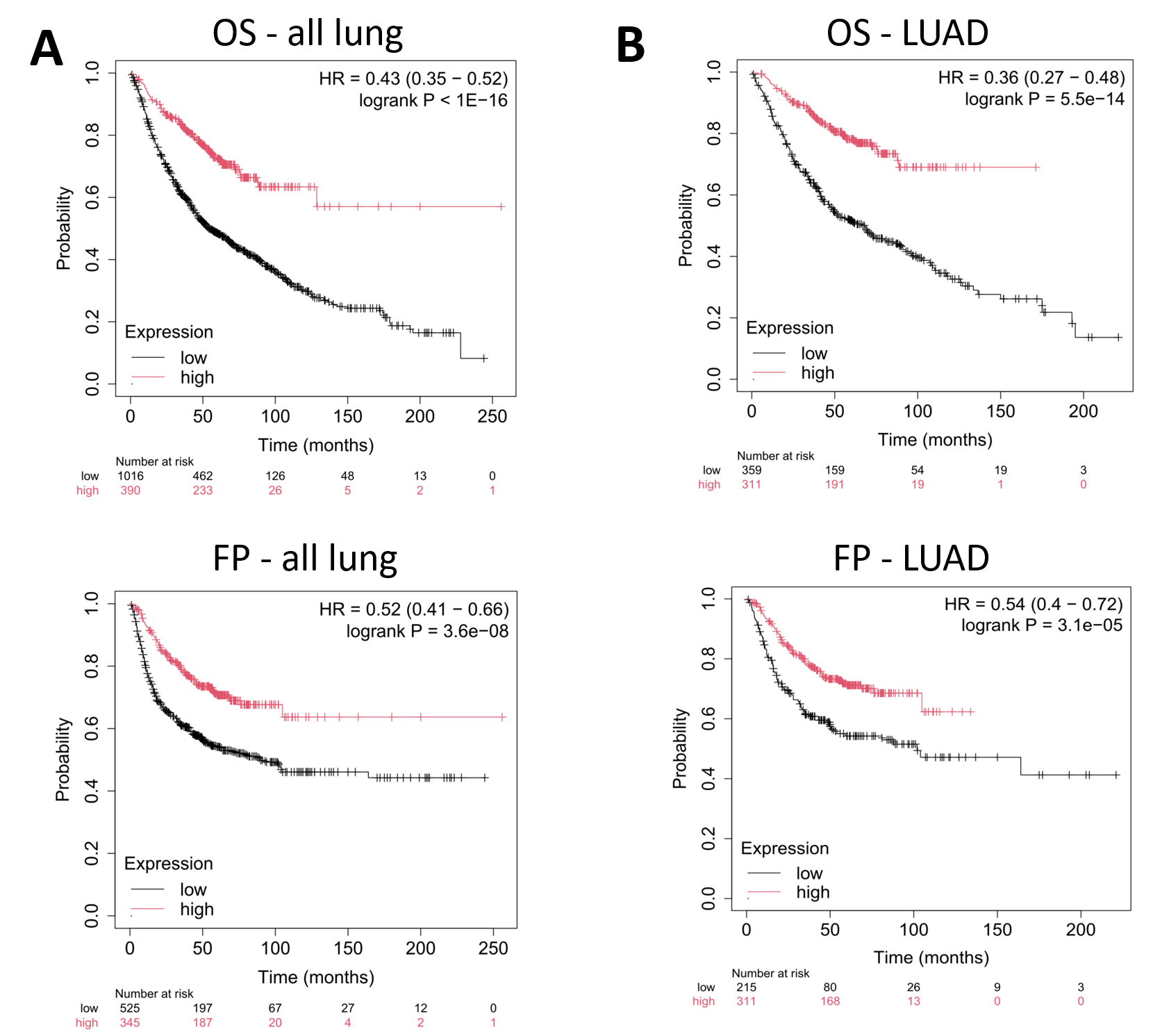


**Supplemental Table 1. Gene lists of the senescence signatures.**

| **SenMayo** | **Li 2025** | **Wu 2023** |
| --- | --- | --- |
| ANGPTL4 | STX3 | SLC9A3R2 |
| BMP2 | ETF1 | S100A16 |
| CCL3 | MAGOH | TNFAIP1 |
| CCL5 | P2RX7 | STC1 |
| CSF1 | CLMP | CAPNS1 |
| CSF2 | PGLYRP4 | TGM2 |
| CST4 | GRINA | FLT1 |
| EGFR | ROBO4 | ITGA5 |
| GDF15 | NDEL1 | PNP |
| GMFG | LAMC1 | ECE1 |
| HMGB1 | NR2F2 | TUBB6 |
| IGFBP2 | RBBP8 | FSCN1 |
| IGFBP3 | FES | RHOC |
| IGFBP4 | PAG1 | YES1 |
| IL10 | SCAMP3 | DOCK6 |
| IL6 | EFNB2 | BCAM |
| INHA | ARHGAP29 | SERPINH1 |
| ITGA2 | TSPAN6 | PLVAP |
| MMP13 | NCKAP1 | LAMA5 |
| PLAU | BMPER | PLEKHG1 |
| RPS6KA5 | GRB10 | TMEM204 |
| SCAMP4 | DLX1 | AFAP1L1 |
| SELPLG | NLGN4X | SYNPO |
| SPP1 | DLL1 | LUZP1 |
| TIMP2 | IL1RL1 | RAB13 |
| TNFRSF10C | HMGB1 | UTRN |
| ACVR1B | PGM2 | FERMT2 |
| ANG | EGFL6 | MYCT1 |
| ANGPT1 | TJP3 | NES |
| AREG | FZD4 | MMRN2 |
| AXL |  | TCF4 |
| BEX3 |  | MAGI1 |
| BMP6 |  | MAST4 |
| C3 |  | PLXND1 |
| CCL1 |  | PHACTR2 |
| CCL13 |  | PLXNA2 |
| CCL16 |  | EFNB2 |
| CCL2 |  | COL4A2 |
| CCL20 |  | COX7A1 |
| CCL24 |  | CD34 |
| CCL26 |  | NID1 |
| CCL4 |  | DYSF |
| CCL7 |  | LAMB1 |
| CCL8 |  | LAMC1 |
| CD55 |  | CD93 |
| CD9 |  | HECW2 |
| CSF2RB |  | FZD4 |
| CTNNB1 |  | BMPR2 |
| CTSB |  | MECOM |
| CXCL1 |  | RGS3 |
| CXCL10 |  | VWA1 |
| CXCL12 |  | CAV2 |
| CXCL16 |  | RHOJ |
| CXCL2 |  | DUSP6 |
| CXCL3 |  | BCL6B |
| CXCL8 |  | A2M |
| DKK1 |  | SCARF1 |
| EDN1 |  | UACA |
| EGF |  | NRP1 |
| EREG |  | ZEB1 |
| ESM1 |  | PPIC |
| ETS2 |  | ICAM2 |
| FAS |  | KDR |
| FGF1 |  | ACVRL1 |
| FGF2 |  | IGFBP7 |
| FGF7 |  | TRIOBP |
| GEM |  | SLC44A2 |
| HGF |  | GALNT18 |
| ICAM1 |  | PODXL |
| ICAM3 |  | PTPRG |
| IGF1 |  | TM4SF18 |
| IGFBP1 |  | CDH5 |
| IGFBP5 |  | PLK2 |
| IGFBP6 |  | LMO2 |
| IGFBP7 |  | LRRC8A |
| IL13 |  | PLEKHA1 |
| IL15 |  | LAMA4 |
| IL18 |  | DLL4 |
| IL1A |  | ESAM |
| IL1B |  | DLC1 |
| IL2 |  | IL3RA |
| IL32 |  | EFNA1 |
| IL6ST |  | TPM4 |
| IL7 |  | SPARC |
| IQGAP2 |  | PRSS23 |
| ITPKA |  | GRB10 |
| JUN |  | NFIA |
| KITLG |  | JAG1 |
| LCP1 |  | GNAI2 |
| MIF |  | PCDH12 |
| MMP1 |  | GJA1 |
| MMP10 |  | CYYR1 |
| MMP12 |  | COL4A1 |
| MMP14 |  | INSR |
| MMP2 |  | EHD4 |
| MMP3 |  | PEA15 |
| MMP9 |  | MGLL |
| NAP1L4 |  |  |
| NRG1 |  |  |
| PAPPA |  |  |
| PECAM1 |  |  |
| PGF |  |  |
| PIGF |  |  |
| PLAT |  |  |
| PLAUR |  |  |
| PTBP1 |  |  |
| PTGER2 |  |  |
| PTGES |  |  |
| SEMA3F |  |  |
| SERPINB4 |  |  |
| SERPINE1 |  |  |
| SERPINE2 |  |  |
| TNF |  |  |
| TNFRSF11B |  |  |
| TNFRSF1A |  |  |
| TNFRSF1B |  |  |
| TUBGCP2 |  |  |
| VEGFA |  |  |
| VEGFC |  |  |
| VGF |  |  |
| WNT16 |  |  |
| WNT2 |  |  |
